# From bench to bedside: translational insights into aging research

**DOI:** 10.3389/fragi.2025.1492099

**Published:** 2025-01-24

**Authors:** Kanti Bhooshan Pandey

**Affiliations:** ^1^ CSIR-Central Salt & Marine Chemicals Research Institute, Bhavnagar, Gujarat, India; ^2^ Faculty of Biological Sciences, Academy of Scientific and Innovative Research, Ghaziabad, Uttar Pradesh, India

**Keywords:** aging, genomic instability, oxidative stress, biomarkers, interventions, longevity

## Abstract

Aging research has rapidly advanced from fundamental discoveries at the molecular and cellular levels to promising clinical applications. This review discusses the critical translational insights that bridge the gap between bench research and bedside applications, highlighting key discoveries in the mechanisms of aging, biomarkers, and therapeutic interventions. It underscores the importance of interdisciplinary approaches and collaboration among scientists, clinicians, and policymakers to address the complexities of aging and improve health span.

## Introduction

Aging is a ubiquitous, multifactorial process marked by a steady loss in physiological processes and an increased vulnerability to illnesses. Researches to understand the aging process and to combat its deteriorative consequences had a long history and perhaps associated with human evolution. During last 100 years significant advances have been seen in exploration of aging process, its regulatory mechanisms, genetic and epigenetic factors, identifying potential biomarkers and screening antiaging drug candidates (Pandey and Rizvi, 2010; Klimova et al., 2018; Guo et al., 2022; Calabrese et al., 2024).

Research on aging began with studies on light intensity and lifespan in *Drosophila* (Northrop, 1925) and progressed to caloric restriction’s impact on longevity (McCay et al., 1975), laying the foundation for modern translational aging studies. After that a number of significant studies on aging process, models of aging, lifespan prolongation and promoting healthy aging were carried out, however, the isolation of longevity mutants in *C. elegans* evoked a new era in aging studies (Klass, 1983). It was finally proven by decades of intensive research work by researchers that the process of aging is multifactorial and linked with many chronic diseases such as cardiovascular disorders, cancer, neuro-complications and diabetes (Guo et al., 2022; Li et al., 2024a).

Aging affects individuals, families, and society both financially and mentally. The UN expects the global population of those aged 65 and older to reach 1.5 billion by 2050 (United Nations, 2019: World Population Ageing 2019: Highlights). With the global population aging rapidly, understanding the biological underpinnings of aging and translating these insights into clinical interventions have become a crucial public health priority. This review aims to synthesize recent advancements in aging research, focusing on translational aspects that hold promise for extending longevity and raising the standard of living of the elderly.

## Mechanisms of aging: from basic research to clinical relevance

Aging is leading risk factors for many chronic conditions such as diabetes, heat disease, neuro-pathologies, cancer, osteoporosis and chronic obstructive pulmonary disease (Guo et al., 2022; Santos et al., 2024). Understanding the mechanisms behind aging is essential for developing effective therapeutic targets. Despite numerous theories attempting to explain this complex biological process, none fully captures the precise mechanisms of aging.

Cellular senescence is one of the most discussed biological processes behind aging. It is a hallmark of aging and reported as a state of irreversible growth arrest. The ability of cells to proliferate and differentiate, as well as their physiological capabilities, gradually declines with time. Both diseases and the preservation of normal tissue homeostasis are significantly impacted by cellular senescence. It speeds up the aging process and the emergence of disorders linked to aging. Senescent cells secrete pro-inflammatory cytokines, contributing to tissue dysfunction. Recent studies have identified senolytic agents, such as Dasatinib and quercetin, which selectively eliminate senescent cells. Preclinical models have shown that senolytics can mitigate age-related pathologies and may extend lifespan, leading to ongoing clinical trials assessing their safety and efficacy in humans (Hickson et al., 2019; Islam et al., 2023).

Extract of aging studies performed on humans and other related model systems, proposes some common events involved in the aging process. Broadly divided in cellular and molecular levels, these features incorporate cellular senescence, weary stem cells, malfunctioned intercellular communications, telomere shortening, genomic instability, proteostasis loss, mitochondrial dysfunction, epigenetic alterations and compromised autophagy (López-Otín et al., 2013; Guo et al., 2022; Boccardi and Marano, 2024) Figure 1. It has also been reported that though numerous intricate and significant pathways contribute to the aging process, many of these processes are linked to chronic oxidative stress brought on by high reactive oxygen species (ROS) levels (Pandey and Rizvi, 2010; Mossad et al., 2022; Yang et al., 2024). This review summarizes major molecular and cellular events that could contribute to a better understanding of the various molecular signalling networks that are involved in an organism’s aging process.

**FIGURE 1:**
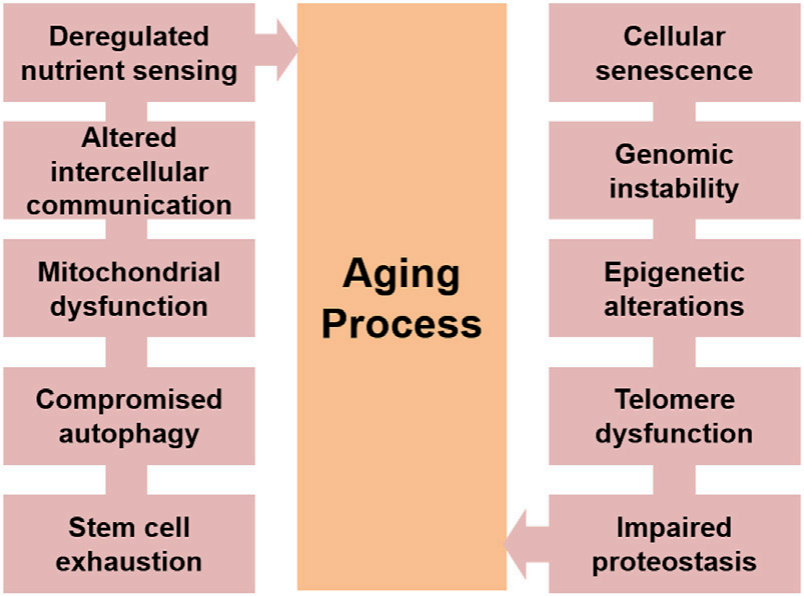
The major hallmarks of aging process.

### Genomic instability

It is reported that cellular senescence is highly promoted by genomic instability of the organism (Aguilera and Garcia-Muse, 2013). Instability in the genome due to many of the reasons such as damage of nuclear DNA (nDNA) and their accumulation pushes the deregulation of gene expression that results in hindrance in the cell growth followed by cell death that leads to aging (Siametis et al., 2021). Damage in nDNA overtime, activates the p53-p21 and p16INK4a-pRb cell cycle checkpoint pathways and eventually prohibits the transmission of genetic information (Williams and Schumacher, 2016; Gorini et al., 2020). Mitochondrial DNA (mtDNA) are again mutation susceptible and there are studies that report that leakage in mitochondrial electrons electron transport chain induces mtDNA to mutate (Kauppila et al., 2017; Son and Lee, 2019; Madreiter-Sokolowski et al., 2024).

Elevated copies of mutated mtDNA and decreased mtDNA copies generate genomic instability in organisms, which drives them towards aging. Moreover, release of mtDNA in cytoplasm under influence of oxidative stress tends to participate in cellular senescence by binding with cyclic guanosine monophosphate-adenosine monophosphate synthase (cGAS) and triggering the stimulator of interferon genes (STING) (Loo et al., 2020). Circulating mtDNA has also been reported to be associated with neuro-diseases, the prominent disorder associated with aging (Reid et al., 2023).

Aseptic inflammation arises when there is no pathogenic infection, and endogenous cellular DNA plays a role in this. Endogenous cellular DNA is also linked to the onset of other chronic illnesses, such as neurological problems and cardiovascular diseases. Recent studies have also suggested that endogenous cytoplasmic DNA and non-protein encoding DNA (junk DNA) also contribute to genetic diversity of aging via affecting telomerase gene activity and aseptic inflammation by mediating CD^4+^T proliferation and activation (Wang et al., 2021; Guo et al., 2022).

### Telomere and mitochondrial dysfunction

Telomeres are designed to maintain the genomic stability, however shortening of telomere to the Hayflick limit during cell divisions, causes a DNA damage response, which in turn persuades cell cycle arrest and pro-inflammatory factor expression, ultimately leading to organismal aging (Zhu et al., 2019; Boccardi and Marano, 2024). Telomerase are responsible for the telomere length, and it has been proven that increase in the expression of telomerases increases the lifespan of an organism (Zhu et al., 2019; Howard et al., 2021). Studies have reported a significant downfall in the telomerase activity in elderly in comparison to the embryonic stage activity (Blackburn et al., 2015; Boccardi and Marano, 2024). Interestingly, telomerase can be reactivated in cells in order to mitigate the deteriorating effects of aging when telomere reaches a critical limit of reduction (Sun et al., 2019).

The anti-aging role of telomerases is evidenced by the fact that the rate of DNA damage is higher in skin cells, which do not possesses telomerases than the cells who have telomerases such as embryonic, immune and stems cells (Sun et al., 2019). Overexpression of telomerases are also reported in cancer cells and it may be a protective mechanism to restore the genomic stability to counteract carcinogenesis (Chakravarti et al., 2021). The role of mitochondria is reported to implicate in aging due to their susceptibility to damage and reduced function over time. Interventions targeting mitochondrial biogenesis, such as NAD^+^ precursors (e.g., nicotinamide riboside), have shown promise in improving mitochondrial function and delaying aging-related diseases in animal models (Sharma et al., 2023; Li et al., 2024b). Clinical trials are now evaluating these compounds’ potential to enhance human health span (Lanza and Nair, 2010; Caicedo and Singh, 2023).

### Epigenetic alterations

Aging is associated with changes in DNA methylation, histone modification, and chromatin structure. These epigenetic alterations influence gene expression and cellular function. One of the primary epigenetic modifications in mammals is methylation at cytosine’s fifth position (5 mC) in DNA. Furthermore, the ten-eleven translocation protein family converts 5-methylcytosine to 5-hydroxymethylcytosine (5hmC), a persistent DNA base alteration. These two alterations serve as epigenetic markers (Lopez et al., 2017; Sen et al., 2016). Age dependent decrease in methylation process has been reported and this correlation is being used in development of epigenetic clock, which may determine the biological age, predict possibility of development of age-associated pathologies and may propose the lifespan of the organism (Shireby et al., 2020; Fiorito et al., 2021; Crofts et al., 2024).

Although different histone modifications occur, acetylation and methylation are the significant modifications associated with cellular senescence. Methylation of histone 3 has different effects and its function depends on the methylation sites and types as well (Padeken et al., 2022). An array of studies have reported that histone acetyltransferases and deacetylases associated with histone modification, play key role in life span determination of an organism that further provides a prominent strategy for antiaging therapies based on histone modification (Yi and Kim, 2020; Padeken et al., 2022). Compromised chromatin structure, polarity and integrity has been reported in the epigenomes of senescence cells, which severely affects chromatin accessibility (Liu et al., 2022).

Transcriptional errors have also been reported during the aging process. Studies have documented that single stranded as well as double stranded RNAs (dsRNA) generated from the unstable genome are closely related with senescence (Saldi et al., 2019; López-Gil et al., 2023). Zhang and co-workers in their study on hypothalamic stem cells have reported that content of exosomal miRNAs secreted by hypothalamic brain stem/progenitor cells declines during aging, while the aging progress significantly delayed after treatment with exosomes secreted by healthy hypothalamic brain stem/progenitor cells (Zhang et al., 2017). Another study performed by Salidi and colleagues documents that chromatin alteration, epigenetic modifications have been associated with age-induced neuro alterations, and proteins associated with neurodegeneration are directly related with derepression of repetitive element transcription due to changes in heterochromatin. Due to this derepression, intracellular dsRNA accumulates more and more, which triggers innate immune responses and adds to the neuroinflammation seen in nearly all age-related neurodegenerative disorders (Saldi et al., 2019). Reprogramming technologies, such as partial reprogramming using Yamanaka factors, have demonstrated the potential to reverse some aspects of cellular aging *in vitro*. Translating these findings into therapeutic strategies requires careful consideration of safety and the risk of oncogenic transformation.

### Impaired proteostasis

Proteins inside human body need to remain folded in a particular manner throughout their lifetimes to perform their biological activities for which they are designed, irrespective of influence of different extra and intracellular stressors. For a successful physiology this protein biome; proteome must be controlled very finely and this balance it termed as proteostasis. In addition to the preventing the protein from misfolding, the proteostasis also responsible for the removal of the misfolded proteins either by autophagy or proteome-mediated degradation. Failure of this homeostasis is associated with health ailments (Hipp et al., 2019; Tang and Xiao, 2023).

Protein misfolding and their accumulation are well documented during aging. Most of the reported age-dependent chronic diseases including neurological events are associated with proteostasis loss (Hipp et al., 2019; Sabath et al., 2020). Cells undergo adaptive modifications when their proteostasis is disrupted and develop a variety of methods to minimise misfolding and eliminate misfolded proteins in order to deal with such adverse circumstances. The synthesis of chaperones is one of the many proteostasis preventive mechanisms, which bind to incomplete peptide chains and help the peptides fold into the proper shape by preventing them from folding too early. These proteins are also known as heat shock proteins because chaperones lessen the denaturation of proteins that happens when cells undergo heat shock (Koyuncu et al., 2021).

A compromised proteostasis has been reported during aging due to elevation in accumulation of misfolded proteins or oxidised proteins under influence of stressors or weakened preventive mechanisms (Powers et al., 2009; Pandey et al., 2010; Hipp et al., 2019). There is evidence that some aged organisms show overexpression of molecular chaperons including small heat-shock proteins or Hsc70 to prevent accumulation of misfolded proteins and their (Söti and Csermely, 2002). However, a multitude of data indicate that the induction of different chaperones is compromised in older organisms, which further promote protein misfolding followed by accumulation (Calderwood et al., 2009; Brehme et al., 2014; Ruano, 2021).

Unfolded protein response (UPR) is cellular stress response that works to enhance the protein folding capacity of endoplasmic reticulum as well as mitochondria. However, once the alignment among protein degradation, misfolding, recycling and UPR is disordered, the loss of proteostasis eventually occurs (Zhou et al., 2021). It has been reported that activating transcription factors 3 and 4 (ATF3 and ATF4), that regulate UPR, play a crucial role in the aging process (Zhao et al., 2021).

Studies have provided experimental evidence that the ability of an organism to maintain proteostasis strongly correlates with the life as well as health spans (Hipp et al., 2019) which advocates the proposal that the adoptions to restore/maintain proteostasis may prolong life span. This is supported by the reporting of life span elongation of *C. elegans* by inhibition of insulin/IGI1 signalling pathway (mutations in the daf-2 gene) (Kenyon et al., 1993; Kenyon, 2011).

## Biomarkers of aging: towards precision medicine

It was 1988, when the first time the idea of ‘a biomarker of aging’ was proposed, defining it as a biological measure of an organism to predict functional capability (Baker and Sprott, 1988). Identifying reliable biomarkers of aging is essential for diagnosing age-related conditions, monitoring therapeutic responses, and predicting individual health trajectories. Since last few decades, various biomarkers of aging have been suggested, encompassing molecular alterations, imaging features, and clinical manifestations, however taking together the heterogeneity of aging, identifying reliable and substantial biomarkers of aging are essential for precise risk assessment of individuals and antiaging interventions. It is interesting that no biomarker yet is documented that qualifies all the desired criteria to track the aging process; the efforts have focused on developing composite biomarkers. This review provides an overview of some reproducible biomarkers of aging based on biological mechanisms underlying the aging process in humans.

**FIGURE 2:**
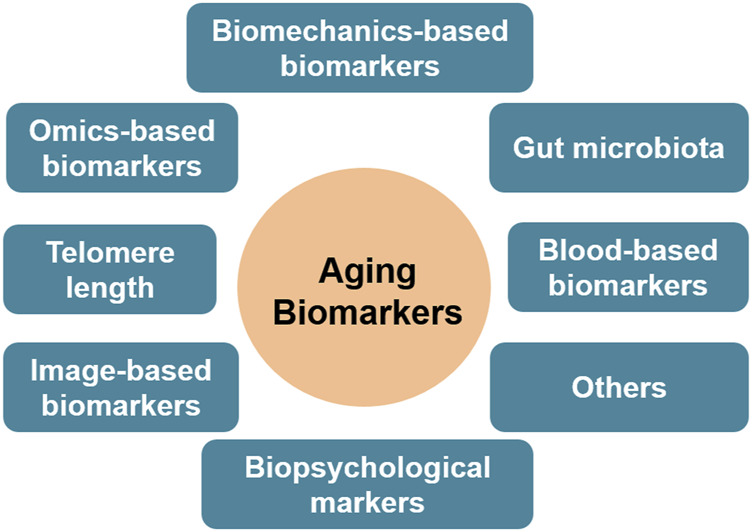
Biomarkers of aging, based on the data sources utilized.

No any gold standard method/criteria has been established to determine the biomarkers of aging. Based on the biological events involved in the aging process, some qualifying biomarkers are summarised (Figure 2). As discussed in the mechanisms section, telomere can protect DNA from damage and instability. After each cell cycle, the telomeres in the majority of somatic cells shorten by 50–150 base pairs (Griffith et al., 1999). Given that most human somatic cells divide only a limited number of times before their protective capacity is depleted and thus become susceptible to mutations, telomere length is thought to be a biomarker of aging. Studies have revealed that the majority of cells and tissues, including fibroblasts, peripheral blood cells, and the mucosa of the colon, had a predictable reduction in telomere length with aging (Tsuji et al., 2002; Ren et al., 2009; Chen et al., 2023).

DNA methylation establishes the epigenetic clock that controls the transcription of genes. Because CpGs methylation status changes at distinct places in the genome with age, DNA methylation status is significant in biomarking of age (Horvath and Raj, 2018). Additionally, it has been shown that millions of CpGs have a role in aging, and an increasing amount of data indicates that DNA methylation status has a substantial correlation with both chronological age and age-related pathologies (Alisch et al., 2012; Zhang et al., 2019; Yasmeen et al., 2020).

Transcriptomics is another new study area to biomark aging. An array of reports from different aging models systems and laboratories has shown age-related alterations in the RNA expression patterns of several organs, such as the kidney, skin, blood, and brain (Zhang et al., 2015). There are experimental evidence that have the measurement of non-coding microRNAs may provide a novel, noninvasive approach for the identification of the aging process, however, larger, more varied longitudinal cohort studies are needed to confirm the validity of transcriptome age in predicting age-specific effects (Zhang et al., 2015; Kinser and Pincus, 2020).

Likewise, proteomics is also an emerging study area to identify aging and associated events. Numerous studies on different model samples such as blood, bone marrow, skin and cerebrospinal fluid have documented that proteins drive aging process (Pandey et al., 2010; Baird et al., 2012; Hennrich et al., 2018; Argentieri et al., 2024). A recent study reports that proteins from a variety of functional categories are involved in proteome aging and this process can be utilized to predict mortality risk, multimorbidity, and age-related functional status in populations with different genetic and geographic backgrounds (Argentieri et al., 2024). However, the proteomic age is still in its infancy despite these encouraging results because of the enormous complexity of the proteome and the absence of standardized testing instruments (Chen et al., 2023).

The systematic study of metabolites may also present the biological age since aging and metabolism are undoubtedly linked since it has been experimentally proved that metabolites are directly involved in the aging process (Menni et al., 2013). The health of gut microbiota is also may also be a mirror of biological age, in the last decade, many studies on changes on gut microbiota and aging have been reported which claim that signify the role gut microbiota in promoting health aging through maintaining gut diversity and homeostasis (Biagi et al., 2013; de Vos et al., 2022).

Blood-based clinical parameters are one of the most authentic and ancient biomarkers of aging and related diseases. An array of studies on human and other aging models has provided that lipid peroxidation and aggregation, levels of glutathione, thiols, ascorbate recycling, complete blood count, alanine aminotransferases (ALT), aspartate aminotransferases (AST), γ-glutamyl transferases (GGT) and other liver enzymes, increased creatinine content and impaired glomerular rate and immune profiles are significant biomarkers of prevalence of oxidative stress and aging (Pandey and Rizvi, 2010; Pandey and Rizvi, 2015a; Cieslak et al., 2016; Mamoshina et al., 2019; Yousefzadeh et al., 2021).

Apart from the above-discussed area that may biomark aging, the concept of image-based aging biomarkers is also proposed based on the correlation between the structure and the functions of the aging organs such as face, retina, brain, heart. Neuroimaging methods like positron emission tomography (PET) and magnetic resonance imaging (MRI) provide a special window to determine neural aging by identifying grey matter and cerebrospinal fluid, white matter connectivity, cortical thickness (MacDonald and Pike, 2021). Optical coherence tomography (OCT) by capturing 3D images of retina at micrometre level provides optical biopsy of vascular and neuroanatomical changes that may be used to predict the biological age (Zhu et al., 2022; Chen et al., 2023). 3D facial images and ECG leads have also been claimed to mark the biological age by different researchers (Chen et al., 2015; Ball et al., 2014), however, further studies are needed to validate these documentations.

Although, the existence of reliable aging biomarker(s) are still under debate, but explored biomarkers so far have been used in precision medicine in the ageing world. Preventive pathways of the aging process such as reducing the inflammation, restoration of redox status, diminishing the aberrant immune responses and enhancing the antioxidant potential are current strategies based on the identified biomarkers. In high risk populations, the FDA-approved preventive medications used for common diseases such as diabetes and cardiovascular ailments are already widely being used as an anti-ageing tool such as like metformin, rapamycin, doxazosin, and so forth (Barzilai et al., 2016; Ettehad et al., 2016; McIntyre et al., 2021; Chen et al., 2023).

## Therapeutic interventions: current strategies and future directions

Significant findings in the last 3 decades have provided possibilities to intervene in the aging process at least to promote healthy aging. Prolongation of life span is still a hard nut to crack. Rapamycin, an inhibitor of the mTOR pathway, has shown efficacy in extending lifespan in various model organisms. Clinical trials are exploring its potential in improving immune function and reducing the incidence of age-related diseases in humans (Papadopoli et al., 2019; Lee et al., 2024). Metformin, which is originally used for diabetes management, has been found to be associated with reduced incidence of carcinogenesis and development of cardiovascular ailments. Ongoing trials, such as the TAME (Targeting Aging with Metformin) study, are investigating its broader applications in aging (Barzilai et al., 2016; Kulkarni et al., 2020).

Dietary intervention and change in lifestyle is one of the most advocated antiaging treatments since years back. Polyphenols, the secondary metabolites that naturally occur in plants and plant-products are reported to combat aging consequences through diminishing the cellular molecular processes involved in aging (Pandey and Suttajit, 2022). Adherence on Mediterranean Diet (MD) which is characterized by a high intake of fruit, vegetables, breads, other cereals, beans, nuts, and seeds has also been reported to slow down the onset of aging and related pathological events (Pandey, 2018; Capurso et al., 2019). Caloric restriction and intermittent fasting have been shown to extend lifespan and improve metabolic health in animal models (James et al., 2024). Regular physical activity is well-documented to enhance health span by improving cardiovascular health, muscle function, and cognitive abilities. Research is ongoing to optimize exercise regimens tailored to older adults (Pandey and Rizvi, 2015b; Ho et al., 2022). Translating these findings into human practice involves balancing benefits with potential risks, and understanding individual variability.

Regenerative medication is another recent area in antiaging research. Therapies based on stem cells offer potential for regenerating damaged tissues and organs. Advances in induced pluripotent stem cells (iPSCs) and mesenchymal stem cells (MSCs) are paving the way for clinical applications in treating age-related degenerative conditions (Aboul-Soud et al., 2021). Bioengineering techniques are being developed to create functional tissues and organs *in vitro*. These technologies hold promise for addressing organ failure and other age-related tissue dysfunctions.

However, despite remarkable progress in understanding the aging process and its possible intervention, many challenges remain in translating aging research into clinical practice and filling the gap between bench research and bedside applications. In a similar vein, more thorough comparative studies of aging are still needed in order to facilitate precise translational research. It is very crucial to understand that all the aging models have limits and this may enhance translational difficulties (Sukoff Rizzo et al., 2023). Researchers in this area must be cautious to avoid misinterpreting the results and have reasonable expectations on the translatability of preclinical findings. For example, despite encouraging results in rodent models in preclinical studies, there has been a major translational failure in human studies, and the results have frequently been over-interpreted (Silverman et al., 2020; Muratoglu et al., 2022). A closer collaboration between basic scientists and clinical researchers is highly recommended for effective translational outcomes.

Other challenges in translational ageing research include ensuring the safety and efficacy of new interventions, addressing ethical and regulatory concerns, and managing the high costs associated with advanced therapies. Keeping this rationale in mind, future research should focus on integrative approaches by combining metabolomics, proteomic, transcriptomic, genetic and epigenetic data to design a comprehensive approach and personalized therapies and global collaborations to share knowledge, standardize protocols, and accelerate the development of aging interventions.

## Conclusion

In the field of aging research, there are important obstacles as well as encouraging findings along the way from bench to bedside. Through interdisciplinary approaches and collaborative efforts, we can expedite the conversion of scientific discoveries into efficacious therapeutic interventions. However, breakthroughs are anticipated in many areas such as exploring common and different aging mechanisms in different human races, investigating potential and reliable biomarkers by fusing data from various sources or utilizing new technologies including artificial intelligence (AI), deep learning and machine learning, and confirming the clinical utility of both established and emerging developing biomarkers.

In addition, DNA methylation age estimators across species and using the closer vertebrates as model systems may provide better understanding of epigenetic aging, resulting in development of precise and safer anti-aging interventions. Currently the anti-aging interventions used are mostly symptomatic and in general adopted after onset of the disease. While reliable intervention needs to target the cause of aging. However, new avenues for treating aging-related diseases are opened up by research on the newest therapeutic strategies, including senescent cell removal, transplantation of stem cells, promoting the expression of anti-aging factors and suppressing the expression of pro-aging factors, and tissue or organ regeneration. Furthermore, technological advancements and interdisciplinary approaches including AI based data interpretation, nanotechnology based drug delivery, therapeutic antibodies have unlimited possibility to explore aging mechanisms and precise interventions as well. Interestingly some of the above mentioned strategies have been applied in clinical trials and some are under study, however, long-term research is required to validate the effectiveness of these therapies. In the end, these initiatives will open the door to a time when aging is a stage of sustained health and vitality rather than just a loss of function.
